# Impact of Auditory Context on Executed Motor Actions

**DOI:** 10.3389/fnint.2016.00001

**Published:** 2016-01-19

**Authors:** Michal Yoles-Frenkel, Maayan Avron, Yifat Prut

**Affiliations:** Department of Medical Neurobiology, Institute of Medical Research Israel-Canada, Edmond and Lily Safra Center for Brain Research, The Hebrew UniversityJerusalem, Israel

**Keywords:** rhythmic movements, auditory–motor interactions, muscle activity, EMG, tapping

## Abstract

The auditory and motor systems are strongly coupled, as is evident in the specifically tight motor synchronization that occurs in response to regularly occurring auditory cues compared with cues of other modalities. Timing of rhythmic action is known to rely on multiple neural centers including the cerebellum and the basal-ganglia which have access to both motor cortical and spinal circuitries. To date, however, there is little information on the motor mechanisms that operate during preparation and execution of rhythmic vs. non-rhythmic movements. We measured acceleration profile and muscle activity while subjects performed tapping movements in response to auditory cues. We found that when tapping at random intervals there was a higher variability of both acceleration profile and muscle activity during motor preparation compared to rhythmic tapping. However, the specific rhythmic context (cued, self-paced, or syncopation) did not affect the motor parameters of the executed taps. Finally, during entrainment we found a gradual as opposed to episodic change in low-level motor parameters (i.e., preparatory muscle activity) that was strongly correlated with changes in high-level parameters (i.e., shift in the reaction time to negative asynchrony). These findings suggest that motor entrainment involves not only adjusting the timing of movement but also modifying parameters that are related to its production. These changes in motor output were insensitive to the specifics of the rhythmic cue: although it took subjects different times to become entrained to different types of rhythmic cues, the motor actions produced once entrainment was obtained were indistinguishable. These findings suggest that motor entrainment involves not only adjusting the timing of movement but also modifying parameters related to its production. The reduced variability of muscle activity during the preparatory period could be one mechanism used by the motor system to enhance the accuracy of motor timing.

## Introduction

The auditory and motor systems are tightly coupled via direct and indirect anatomical pathways. The functional implications of this coupling range from the primitive startle reflex (Yeomans and Frankland, 1995) to complex movements that follow rhythmic cues. The cerebellum is hypothesized to be an internal timing system in the milliseconds to second range, (Ivry, 1997; Ivry et al., 2002; Ivry and Spencer, 2004; Del Olmo et al., 2007). In addition, other neural systems, such as the Basal Ganglia (Buhusi and Meck, 2005; Jin et al., 2009) and prefrontal cortical areas (Zarco et al., 2009; Narayanan et al., 2013) have been shown to contribute to timing of actions, and are thus likely to contribute to rhythmic tasks performance.

A common paradigm to study motor entrainment to rhythmic cues is finger tapping: a simple discontinuous rhythmic task through which auditory-motor coupling can be probed (Michon and van der Valk, 1967). In this task, motor synchronization to the auditory cue occurs very rapidly. Within 2–3 stimuli, subject tapping-times become predictive so that the tap occurs several tens of milliseconds before the auditory cue is delivered. This property, which is often referred to as negative asynchrony (Aschersleben and Prinz, 1995; Miyake et al., 2004; Pollok et al., 2004), shows that the motor command is generated before the onset of the auditory stimulus through an anticipatory timing control (Thaut et al., 1999). While the perceptual aspects of this rhythmic entrainment have been studied extensively (Thaut, 2003; Repp, 2005), much less is known about concurrent changes in motor preparation and motor performance during entrainment and after negative asynchrony is achieved.

Studies of muscle activity during rhythmic movements have shown that when moving in response to auditory rhythmic cues, both the upper and lower limbs exhibit a different electromyogram (EMG) pattern than during self-paced rhythmic movements (Safranek et al., 1982; Thaut et al., 1991, 1992). These changes include a decrease in EMG variations and changes in the temporal pattern of EMG activity (Safranek et al., 1982; Thaut et al., 1991, 1992). It was suggested that changes in motor performance (i.e., movement trajectory) might be used for timing control (Balasubramaniam et al., 2004). A systematic comparison of the extent to which low-level motor parameters (e.g., pattern of muscle activity) are modified during motor preparation and execution by the auditory context has yet to be conducted.

The current study was designed to explore the motor consequences of rhythmic entrainment when changing the auditory context. Specifically, we investigated two related questions; first, whether the mechanism that times rhythmic movements could also affect the properties of the action produced, and second, what are the tap-to-tap interactions between the course of entrainment to a rhythmic cue and subsequent actions. We found that rhythmic and random tapping differed substantially in the motor variability measured during the pre-tap period but not during the actual tap. We further confirmed that rhythmic tapping to different auditory cues involves different entrainment processes. Rhythm acquisition was gradual in terms of both the establishment of negative synchronization and the corresponding reduction in motor variability. Nonetheless, the reduced variability observed when entrainment was achieved was insensitive to the specific auditory contexts used to induce rhythmicity.

Our results suggest that high-level cognitive processes such as rhythm perception have ongoing low-level motor correlates manifested as changes in muscle activation patterns. The differences in motor preparation between rhythmic and random tapping support the notion that these actions recruit different neuronal mechanisms (Howard et al., 2011). However, different rhythmic actions appear to use a common motor circuitry, despite the apparent differences in the process of rhythm acquisition.

## Materials and Methods

Thirty four (34) subjects participated in this study. All were healthy and right handed (self-reported) and ranged in age from 22 to 37 (mean = 27.98 ± 3.63 years). Participants gave informed consent to take part in the experiments. The experimental procedure was approved by the ethics committee of the Hebrew University. Each subject was requested to fill in a short questionnaire regarding his/her musical history and personal hobbies.

### Experimental Setup

**FIGURE 1:**
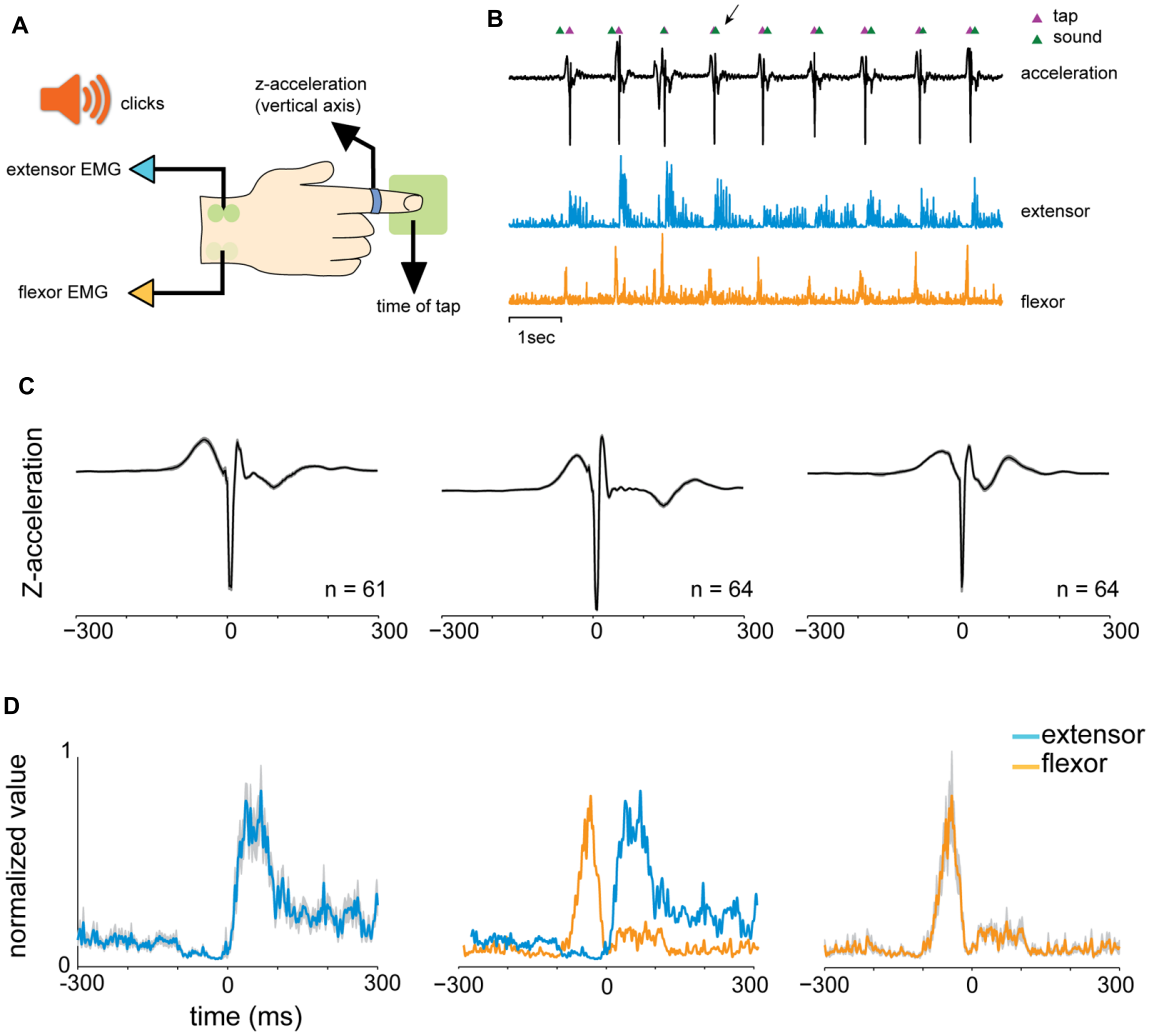
**Experimental design. (A)** Experimental setup: participants sat at a desk, with their right hand on a “tapping surface.” An accelerometer was placed on the index finger immediately above the lower metacarpal joint. Tapping was measured by a proximity sensor that captured the exact time of the tap. Finger movement was constrained only to the vertical axis (*z*-acceleration). Auditory cues (“clicks”) were given through headphones. The EMG of the flexor digitorium superfacialis (FDS) and the extensor indicis proprius (EIP) were recorded using surface electrodes. **(B)** One data block consisted of nine taps. Shown data include the acceleration signal (black), tap events (magenta triangles) detected by the proximity sensor and the timing of auditory signals (green triangles). Muscle activity of an extensor (blue) and flexor (orange) muscles are shown as well (after rectification and downsampling). Arrows indicate the first predictive tap which preceded the auditory signal. **(C)** Examples of mean (black) and standard error of the mean (SEM, gray shading) of acceleration profiles of three different subjects, averaged across different conditions around tap time. The profiles varied across subjects but appeared stable within each single subject. **(D)** Mean muscle activity (and SEM in gray shading) of EIP (blue) and FDS (orange) muscles for one subject. Middle plot shows the relative activation profile of the two muscles around tap time (*t* = 0).

The experimental setup is illustrated in **Figure 1**. Participants set at a desk, with their right hand placed on a “tapping surface.” The forearm rested on a flat surface and the wrist and elbow joints were comfortably secured by adjustable straps. An accelerometer was placed on the index finger immediately above the lower metacarpal joint. Subjects were instructed to tap their index finger to a pacing cue, and to maintain their finger extended during the inter-tap intervals (ITI). A custom-made proximity sensor was used to measure the exact time of the tap. Auditory cues were given through headphones (**Figure 1A**). In order to avoid visual feedback, subjects were instructed to look at the computer screen and not at their hand. A verbal explanation on the structure of the experiment was provided, with no training trials.

### Behavioral Task

Subjects were presented with auditory cues that consisted of a 1 kHz tone lasting 50 ms. Three experimental designs were used (**Table 1**):

**Table 1:** Behavioral setup.

Set	Tapping type	Subjects	Beeps	Signals	Repetitions
A	Random	13	10	Acc, Prox	12
A	Rhythm	13	10	Acc, Prox	12
B	Random	16	30	Acc, Prox, EMG	6
B	Rhythmic	16	30	Acc, Prox, EMG	4
B	Syncopation	16^a^	30	Acc, Prox, EMG	4
B and C	Self-paced	30	-	Acc, Prox, EMG	1
C	Joining the rhythm	15	20^b^	Acc, Prox, EMG	2

*^a^Two subjects whose reaction time (RT) STDs were exceptionally high were eliminated from the analysis.*

*^b^Only the last 10 sounds included tapping*.

#### Tapping Experiment A

This paradigm involved two kinds of trials: the first type of trials was “Rhythmic Tapping” in which subjects were asked to tap in synchrony to an isochronous beat (“on beat”) in frequencies of 1 and 2 Hz. The second type of trials was “Random Tapping,” in which subjects were asked to tap to random auditory tones. Intervals between successive tones were randomly drawn from a uniform distribution with a lower bound of 500 ms and an upper bound of 1500 ms. Successive intervals were never identical to prevent effects of synchronizing during the non-rhythmic (random) tapping.

Each trial was composed of 10 sounds. There were twenty-two trials in each condition (for a total of 44 trials). In this task the acceleration and tapping events (without EMG) were recorded from 13 subjects.

#### Tapping Experiment B

In this set of experiments four kinds of trials were used: the first two trial types were “Rhythmic tapping” and “Random tapping” as described in experiment A. The Third type of trial was “Syncopation tapping,” in which subjects were required to tap between two successive tones (“offbeat”) at frequencies of 1 Hz. We found that 2 Hz syncopation was too fast for most of the subjects, and was therefore not used in this study. The fourth type of trials was “Self-paced tapping” task in which subjects were tapped at a natural comfortable pace without an auditory cue.

Each trial was composed of thirty sounds. Each subject performed six sets of random trials, four set of rhythm trials, four sets of syncopation trials and one set of a self-paced tapping trial. In this experiment the recorded signals included acceleration, tapping events and EMG data from 16 subjects.

#### Tapping Experiment C

In this experiment we used two kinds of trials: the first was “Self-paced tapping” task (as in Tapping experiment B). The second type of trials was “Joining the beat” task in which subjects were asked to listen to isochronous tones at a frequency of 1.5 Hz. They were instructed not to tap until a visual cue (red square on the computer screen) appeared. After 10 beeps (during which the subject remained passive) the visual cue appeared and subsequently 10 additional beeps were presented (to which subject had to respond with a tap).

Tapping experiment C was made up of two kinds of trials: one in which the subject had to join the rhythm, and one self-paced tapping trial. In this paradigm we recorded acceleration, tapping events and EMG from 15 subjects.

In all of the experiments, the beginning of a new trial was indicated by a short set of rapid sounds that the subjects were instructed not to respond to. Note that some subjects took part in more than one experimental paradigm and therefore the total number of subject was smaller than the sum of subjects reported for each experimental paradigm. In each figure caption we specify the experimental setup used for the analyses.

### Data Recording and Acquisition

Data were collected continuously and stored using a multi-channel data acquisition system (AlphaLab, Alpha Omega, Nazareth, Israel). Recorded signals included acceleration, auditory signal, tapping events (generated by the proximity sensor) and EMG data. The sampling rate was 12.5 KHz per channel.

An EMG of the *flexor digitorium superfacialis* – FDS (**Figure 1D**) and the *extensor indicis proprius* – EIP (**Figure 1D**) were recorded with Pre-Amplified Electrodes (Motion Lab Systems, MA-411-002, gain x20, 15 Hz–3.5 Kz) that were placed on the skin overlying the muscle. Signals were further amplified (gain x100, total gain x2000) and digitized online.

### Data Analysis

Data files were converted into Matlab format (Mathworks Inc). EMG signals were then rectified and down-sampled to 500 Hz. We found that movement parameters varied across subjects in terms of amplitude and detailed activation pattern and thus could not be compared directly. To overcome inter-subject differences in EMG levels, we calculated the coefficient of variation (CV = standard deviation/mean) of the acceleration and the EMG signals in each condition for each subject. This measure quantified the variability of the data in a manner that was comparable across subjects. The time-resolved CV was computed by first aligning the data on tap-onset and thus assessed the tap-triggered signal (either acceleration or EMG data). We then measured the mean signal and the standard deviation for each time-bin. The bin-wise division of these two measures produced the time-dependent CV.

To test context-dependent changes in signal variability we used the non-parametric Kruskal–Wallis test and not its parametric equivalent ANOVA because the distribution of the CV data was not necessarily normal.

## Results

### Behavioral Analysis of Finger Tapping

**FIGURE 2:**
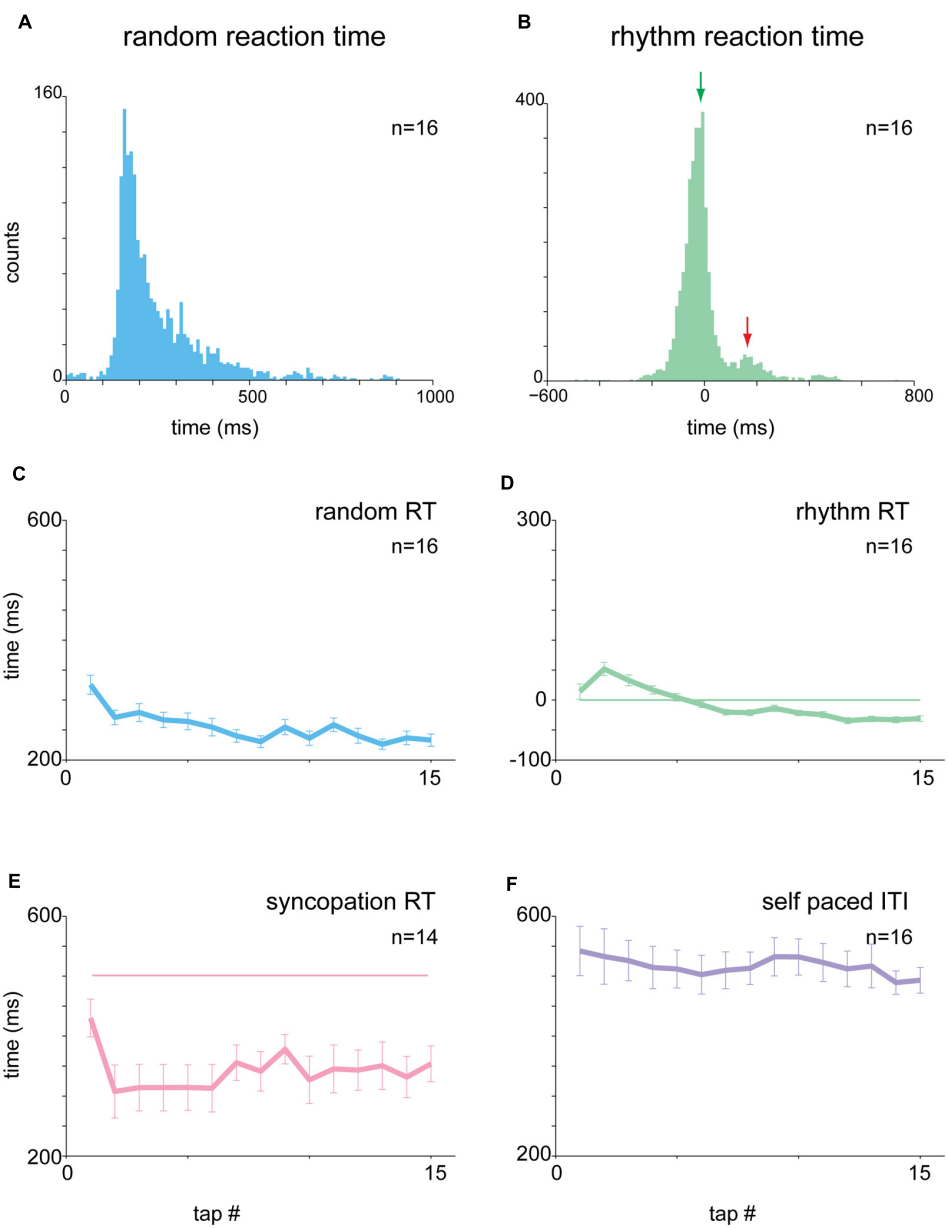
**Analysis of reaction times (RTs). (A)** Distribution of RTs in random tapping. RT was at least +50 ms and exhibited a broad range of values (median = 163.4 ms). **(B)** Distribution of RTs in 1 and 2 Hz blocks. The histogram consisted of two subgroups: a negative asynchrony group (*RT* ≤ 0, green arrow) and a reactive group (*RT* > 0, red arrow). **(C–E)** Tap-dependent modulation of RT in random tapping **(C)**, rhythmic tapping **(D)**, and syncopation trials **(E)**. In **(E)**, the pink horizontal straight line marks the expected interval for syncopation which was 500 ms in the 1 Hz task. **(F)** Tap-dependent modulations of inter tap interval (ITI) in self-paced tapping. Plots showing RT as a function of tap number are not in the same Y-scale, but all have the same range of values (400 ms). The number of subjects is shown for each panel. Note, however, that each subject contributed multiple RT values. Data taken from experimental set B.

We analyzed the reaction times (RTs) of the subjects in the different tapping tasks. In the “random” condition, the auditory cues were presented at random, uncorrelated, intervals. Consequently the distribution of RTs was broad and strictly positive, with RTs exceeding 50 ms (median 163.4 ms, **Figure 2A**). By contrast, RTs measured during rhythmic tapping, when cues were presented at fixed intervals, fell into two different subgroups (**Figure 2B**). The first (and largest) group included predictive taps that preceded the cue by a few tens of milliseconds (green arrow in **Figure 2B**), consistent with a “negative asynchrony” (Aschersleben, 2002). The second group (red arrow in **Figure 2B**) included the reactive taps for which the RT was above 50 ms. This group had a similar time-range as the RTs observed in random tapping and included the first few taps in each set when subjects “acquired” the rhythm but were not fully synchronized to the beat.

Inspecting the time-dependent modulations of RTs along task performance revealed that irrespective of the specific task, RTs slowly stabilized during task performance into a steady mean value. In random tapping, the RT stabilized around a value of 200 ms (**Figure 2C**). In rhythmic tapping, RTs gradually changed from positive (reactive) to negative (predictive) values of -50 ms (**Figure 2D**), when synchronization to the beat was obtained (usually after 3 to 6 taps).

We further tested two additional rhythmic tapping tasks: syncopation and self-paced tapping. In the syncopation task subjects were asked to syncopate; namely tap between two successive sounds. We found that not all subjects were able to perform the task, indicating that syncopation tapping imposes a higher attentional demand on subjects. We thus excluded subjects who exhibited high variability in RTs (measured by the RT standard deviations, 2 out of 16 subjects).

In the 1 Hz syncopation, RTs stabilized at around 350 ms after the sound (**Figure 2E**), a value shorter than half of the inter-sound interval, i.e., 500 ms. In the self-paced tapping subjects were asked to tap with no auditory signals. The mean frequency of this self-paced tapping was 2.07 Hz, a value consistent with previous studies (Yahalom et al., 2004). In this paradigm, due to the absence of auditory cues, RT was undefined and thus behavior was quantified using the ITI (**Figure 2F**). Note that in both syncopation and self-tapping the first few RTs and ITIs were longer and, in the self-paced tapping task, were more variable indicating that here as well a process of “rhythm acquisition” (either externally or internally dictated) took place.

### Acceleration Profiles in Random and Rhythmic Tapping

**FIGURE 3:**
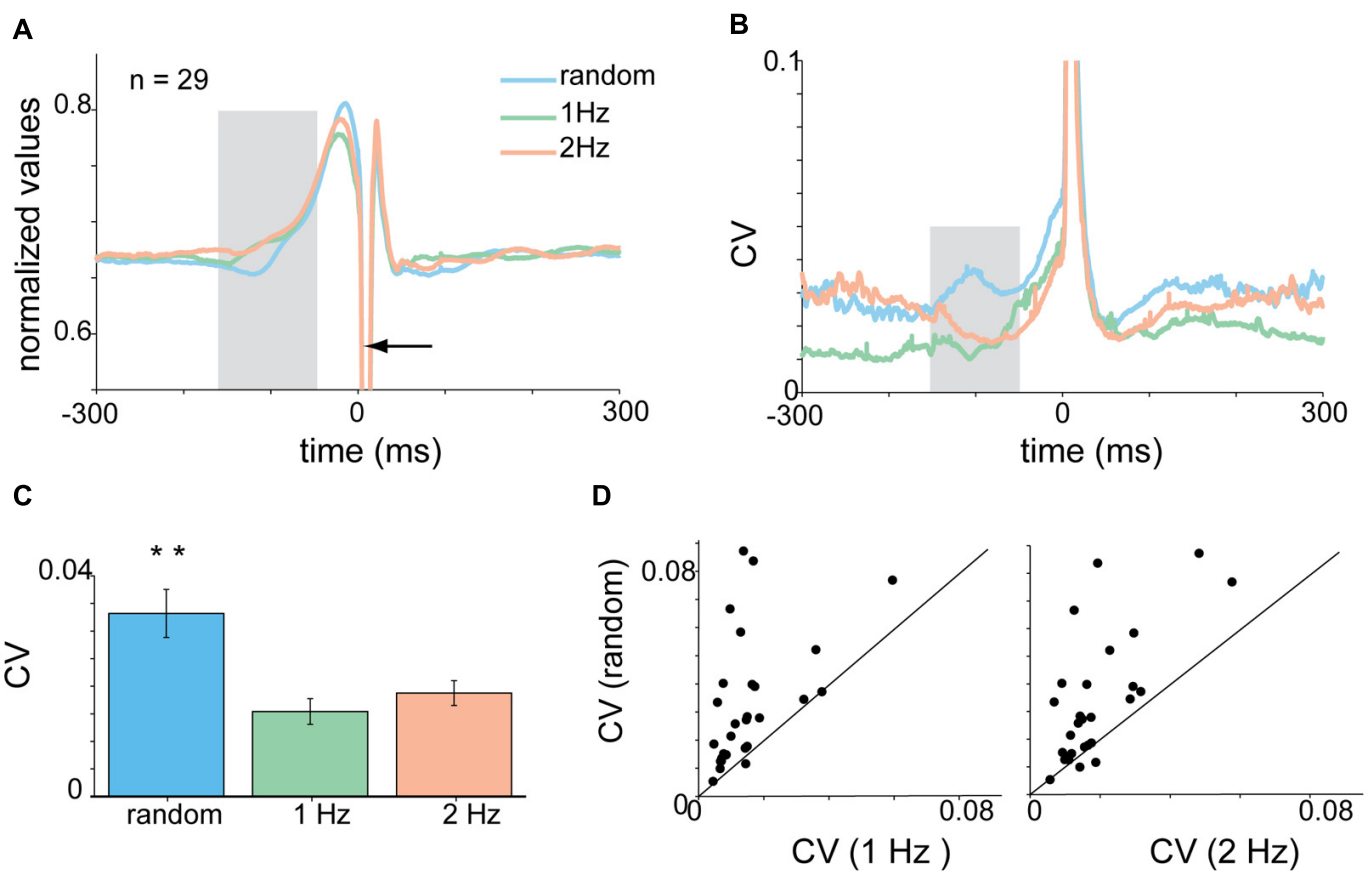
**Context-dependent changes in acceleration profile. (A)** Mean acceleration profile of all subjects (*n* = 28) centered on time of tap. Shaded area corresponds to pre-tap window where random data were significantly different from the 1 and 2 Hz data. **(B)** Same as A, but showing the mean CVt computed for the acceleration signal. **(C)** Mean CV for all subjects computed for the test window (-150 to -50 ms before the tap) during the different task conditions. Mean CV for random tapping was higher than both rhythm condition values (Kruskal–Wallis *p* < 0.01). **(D)** Single subject comparison of CV values found in random vs. rhythmic taps obtained in 1 Hz (left) and 2 Hz (right) tapping. Unity line (*x* = *y*) is shown as well. For the majority of the subjects the values of the random taps were higher than both 1 and 2 Hz rhythmic tapping. Data taken from experimental set A and B. ^∗^*p* < 0.05, ^∗∗^*p* < 0.01.

We studied the task-related motor output by measuring the acceleration profile and muscle activity during task performance. Subjects were asked to maintain their finger between taps in an extension position, to reduce cross-subject variability in tap performance strategy. Consequently, the acceleration profile of a tap typically consisted of a gradual downward acceleration toward the surface followed by a bipolar steep acceleration after the tap itself (finger-touch of the table) and a stop to prepare for the next tap (**Figure 3A**). Note that the steep phases of acceleration and deceleration (arrow in **Figure 3A**) appeared to be the result of the impact with the surface, with no actual finger displacement.

The tap-related acceleration profile varied across subjects but was consistent for each subject across tapping frequencies and conditions. To compare the properties of acceleration across subjects we normalized the signal in the following manner. For each subject we computed the mean acceleration aligned on tap time and then converted it into a time-resolved coefficient of variation (CVt – **Figure 3B**). The averaged CVt obtained in the random and rhythmic conditions was compared using time windows spanning 30 ms and sliding at a 10 ms step. Bins in which significant differences were found between random and rhythmic conditions were marked (*p* < 0.01, Kruskal–Wallis test while adjusting for multiple comparisons). During the tap the CVt measured in the different conditions was not significantly different. Nonetheless, during earlier time bins the CVt diverged, and the random data were more variable (i.e., had a higher CVt) than rhythmic data. To quantify this difference between rhythmic and random tapping we defined the latest 100 ms in which the random and rhythmic tapping were significantly different as an inspection window. For the acceleration data this inspection window spanned from -150 to -50 ms before the time of the tap. In this time window differences in acceleration profile were evident in the mean CV values computed across subjects (**Figure 3C**) as well as when comparing single-subject averaged CV values computed during the same time window (**Figure 3D**). The vast majority of the subjects exhibited higher CV values during the random than in the rhythm conditions (paired *t*-test *p* < 0.0003).

### Context-Dependent Changes in Muscle Activity

**FIGURE 4:**
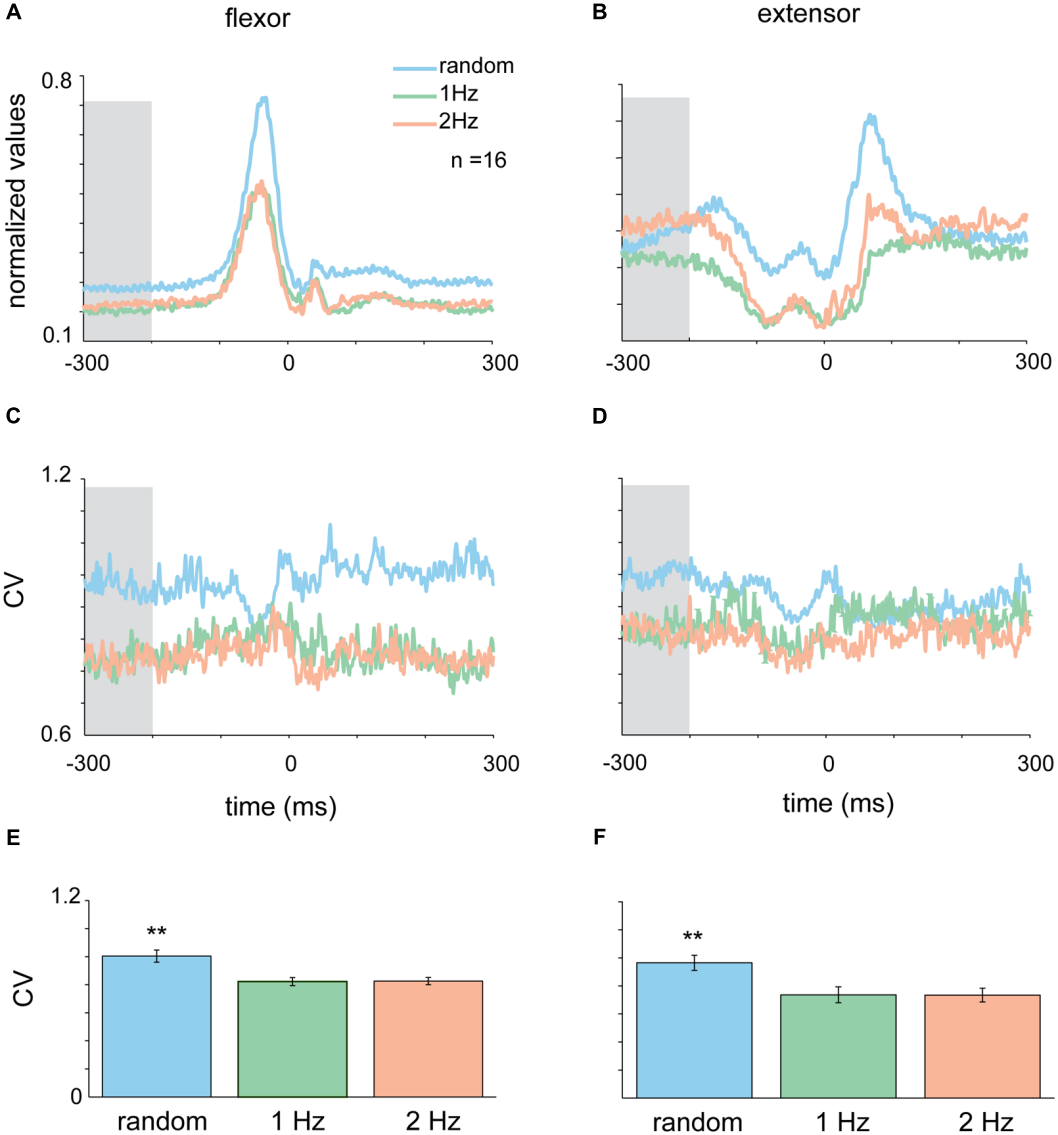
**Context-dependent changes in muscle activity**. Mean EMG for extensor **(A)** and flexor **(B)** muscles in the different tapping conditions aligned on time of tap. Shaded area corresponds to the time-window where variability in the random task was significantly different than variability in the rhythmic task for both flexor and extensor data (-300 to -200 ms before the tap). **(C,D)** CVt of EMG data computed for the random and the two rhythmic conditions. **(E,F)** Mean CV during the test period averaged across all subjects. For both extensor and flexor muscles the CV during random conditions were significantly higher than both rhythmic conditions (Kruskal–Wallis *p* < 0.01). Data taken from experimental set B.

The activity pattern of muscles during the tap was such that flexor muscles were active during the down-phase of the tap when subjects lowered their finger toward the surface (**Figure 4A**), while extensor muscles were active between taps and at the end of the tap when lifting their finger from the surface (**Figure 4B**). Here again we transformed the average muscle activity into CVt and found that the variability of both flexor and extensors muscles during random tapping was consistently higher than the muscle variability during rhythmic tapping (**Figures 4C,D**). We found that the earliest time window in which random tapping was significantly different from rhythmic tapping for both the flexor and extensor muscles occurred -300 to -200 before tap onset, an earlier time window than for the acceleration signal. Within this time window the average CV level of muscle activity in random tapping was significantly higher than in rhythmic tapping for both the extensor muscle (Kruskal–Wallis *p* < 0.0016) and the flexor muscle (Kruskal–Wallis *p* < 0.0061) as depicted in **Figures 4E,F**. Nonetheless, similar to the acceleration signal, after this time window the variability of muscle activity during random tapping was not significantly different from the variability during rhythmic tapping.

### Un-Cued Rhythmic Tapping

The pre-tap reduction in variability of motor activity (i.e., acceleration and muscle activity) in rhythmic tapping could in fact reflect the regular structure of the instructing auditory cue and not a change in pre-movement processing. To test for this option we used two kind of tasks in which tapping was rhythmic but un-cued: self-tapping and syncopation tasks.

**FIGURE 5:**
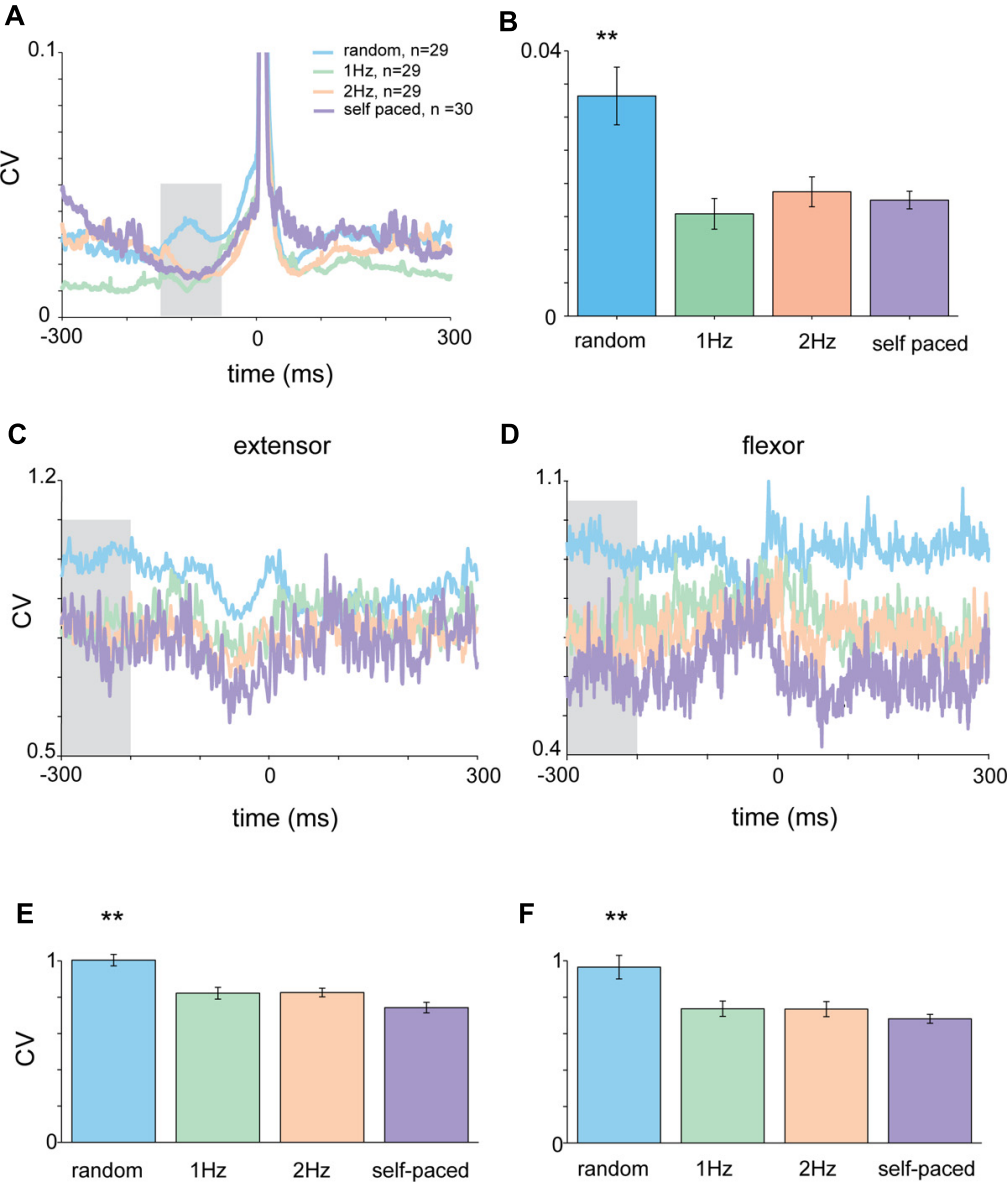
**Acceleration and EMG activity in self-paced tapping. (A)** Mean time-dependent CV of acceleration signal in self-paced, random and rhythmic (1 and 2 Hz) tapping. Shaded area corresponds to the time-window selected for statistically testing the different conditions. **(B)** Average CV during the test period (-150 to -50 ms before the tap) averaged across all subjects (*n* = 30) in the different conditions. Mean CV during self-paced tapping (purple bar) was significantly lower (*p* < 0.01) than random tapping but similar to rhythmic tapping. **(C,D)** CVt of EMG data recorded from extensor **(C)** and flexor **(D)** muscles. Same convention as in **(A)**. **(E,F)** Averaged CV in the inspection window [gray shaded area in panels **(C)** and **(D)**] for the different tapping conditions as measured for extensor **(E)** and flexor **(F)** muscles. Random and rhythmic data taken from experimental set A and B. Self-pace data taken from experimental set B and C. In both cases the CV in random trials was significantly lower (*p* < 0.01) from the CV found in rhythmic and self-paced trials.

During self-paced tapping, the motor parameters of taps were similar to those obtained during cued rhythmic tapping. Specifically, we found a comparable pre-tap reduction in variability of the acceleration profile during the inspection window (-150 to -50 ms, **Figure 5A**) which was significantly different from random, but not cued-rhythmic tapping (**Figure 5B**, *p* < 0.01, Wilcoxon rank sum test). The same result was obtained when analyzing muscle activity during self-paced tapping in the comparable time window (**Figures 5C,D**). The CV of the EMG profile of both the flexor (Kruskal–Wallis *p* < 0.0001) and the extensor (Kruskal–Wallis *p* < 0.00002) muscles (**Figures 5E,F**) in the self-paced tapping was different from the mean CV of the EMG data in the random condition but not in the rhythm conditions (flexor: Kruskal–Wallis *p* = 0.55; extensor: Kruskal–Wallis *p* = 0.08). Thus similar to rhythmic tapping, pre-tap motor variability during self-paced tapping was significantly lower than the pre-tap variability in random tapping.

**FIGURE 6:**
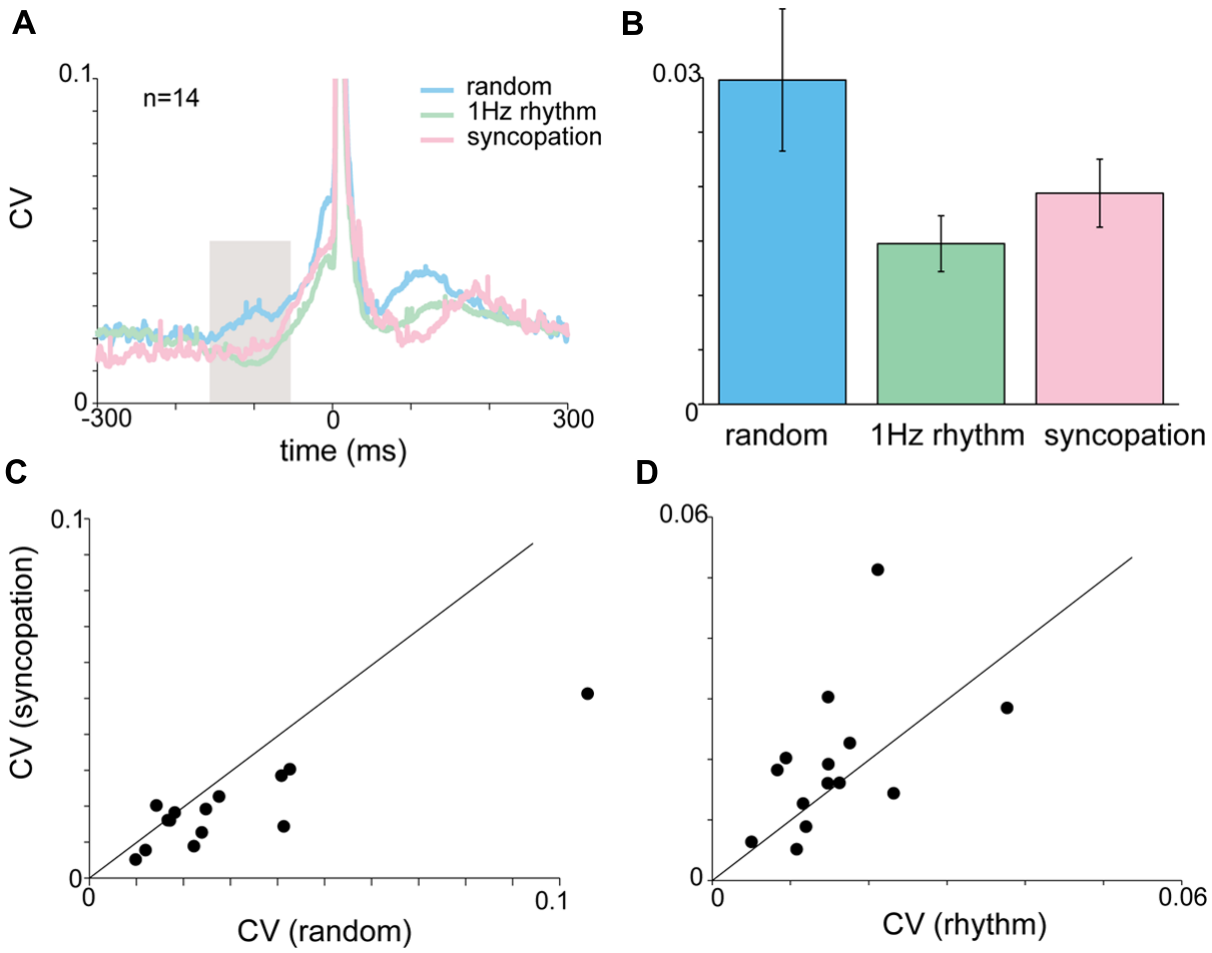
**Acceleration and EMG activity during syncopation task. (A)** CVt of acceleration during random, rhythmic and syncopation tapping. **(B)** Mean CV computed during the inspection window (-150 to -50 ms). No significant difference was found in the mean CV for random, 1 Hz rhythmic and syncopation tasks. Color scheme same as in **(A)**. **(C,D)** Single-subject comparison of the CVs obtained during syncopation with those obtained either during rhythmic tapping **(C)** or random tapping **(D)** revealed significant differences between syncopation and random tapping, but not between syncopation and 1 Hz rhythmic tapping. Data taken from experimental set B after removing two subjects who were unable to syncopate in a stable manner.

During syncopation the auditory cue served as a reference point in time but did not trigger the tap itself. **Figure 6** shows that during syncopation the average variability computed across subjects showed no significant differences from random or rhythm tapping (**Figures 6A,B**). Nonetheless, comparing the single-subject mean CV values (computed by averaging CVt in the inspection window) revealed that at the single-subject level the variability during syncopation trials was consistently lower than in random tapping (**Figure 6C**, paired *t*-test, *p* < 0.023) but not 1 Hz rhythmic tapping (**Figure 6D**). The EMG profile of the syncopation tapping showed a similar pattern. The mean CV of the EMG data (computed across all subjects) during the syncopation trials was similar to the CV level during 1 Hz rhythmic trials and significantly lower than the CV during random trials (Kruskal–Wallis *p* < 0.05).

### The Entrainment Process

**FIGURE 7:**
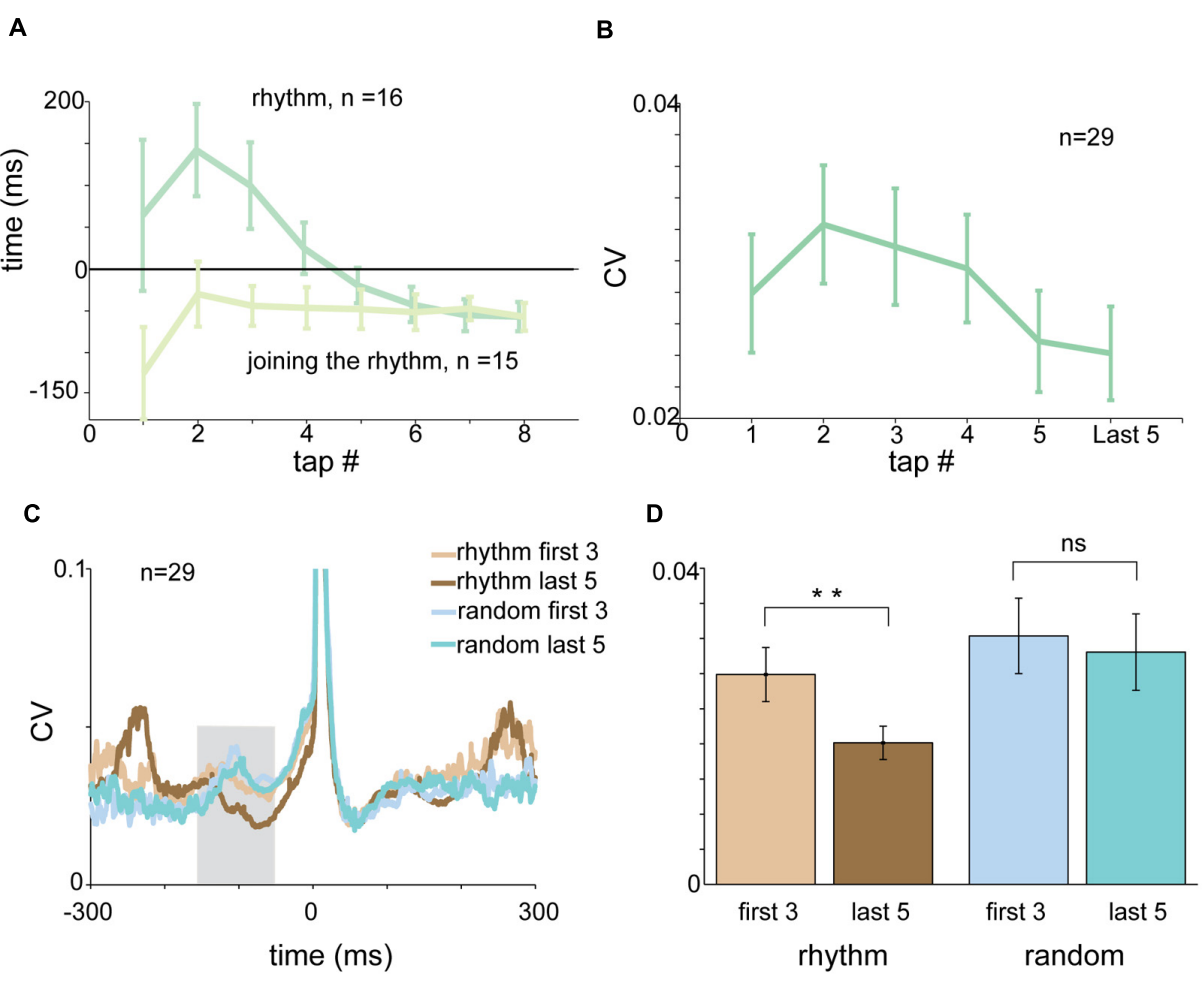
**The entrainment process. (A)** Tap-dependent changes in RT for rhythmic tapping (dark green) and “joining the rhythm” tapping (light green). Negative synchronization was obtained much faster when subjects “joined the rhythm” compared to unprimed tapping. **(B)** Tap-dependent changes in mean CV of acceleration profile computed in a test window spanning -150 to -50 ms before the tap. The CV values decreased gradually and not in an episodic manner. **(C)** CVt of acceleration profile of the first three taps and the last five taps computed for rhythmic tapping (light and dark brown) and random tapping (light and dark cyan). **(D)** Mean CV of acceleration computed for the first three taps (light colors) and the last five taps (dark colors) in rhythmic (brown) and random (blue) conditions. Significant differences were found for rhythmic (paired *t*-test, *p* < 0.01) but not for random tapping. Data for panel **(A)** were taken from experimental set B and C separately. Data for panels **(C,D)** were taken from experimental set A and B.

Each session of rhythmic tapping included an early phase of entrainment during which subjects gradually became synchronized to the beat. In rhythmic tapping the entrainment process was reflected by a gradual shift of the RTs from positive to negative values (**Figure 7A**). The time it took to shift from reactive to predictive tapping depended on the imposed rhythm. In the 2 Hz condition it took on average 2.875 taps (median = 2 taps) until tapping became predictive to the sound. In 1.5 Hz tapping, it took an average of 3.875 taps (median = 3 taps) until tapping became predictive to the sound, and in 1 Hz tapping subjects needed an average of 4.923 taps to become entrained (median = 5 taps).

Asking subjects to start tapping after passive listening to the rhythmic cue greatly accelerated the process of entrainment (defined as the first tap that preceded the sound). Specifically, it took only a single tap until “negative asynchrony” appeared (**Figure 7A**).

We further investigated the changes taking place in the acceleration profile and muscle activity during the entrainment process. **Figure 7B** presents the average CV of acceleration during the pre-tap inspection window (-150 to -50 ms before the tap) as a function of tap number. During this time there was a tap-to-tap reduction in the variability of the signal, suggesting that the entrainment process was gradual rather than episodic. To quantify this process we compared the pre-tap variability of the acceleration during the first three taps to the pre-tap variability during the last five taps of the set (**Figure 7C**). We found that the pre-tap variability of the first three taps in rhythmic tapping was significantly more variable than the corresponding period of the last five taps (paired *t*-test *p* < 0.0079). By contrast, comparing the first three taps to the last five in the random condition revealed no significant differences in the pre-tap variability (**Figure 7D**). The gradual change in pre-tap variability was not observed in the EMG data (for either flexors or extensors), possibly due to the noisy nature of the EMG signal.

## Discussion

The goal of this study was to document the context-dependency of motor parameters during sensorimotor synchronization tasks. We showed that the auditory context (i.e., rhythmic vs. non-rhythmic conditions) substantially affects motor actions, as expressed in reduced variability in both acceleration and muscle activity when preparing for the ensuing tap, but not during the actual tap. We further found that although different kinds of rhythmic tapping (i.e., cued, self-paced and syncopation) took different times to acquire, as soon as synchronization was complete the motor characteristics of the tapping were similar. This similarity was observed irrespective of the fact that in these cases the taps were triggered differently. Finally, we found that the synchronization process is gradual and accompanied by tap-to-tap changes in motor parameters; namely, a reduction in RT and movement variability. Synchronization was also acquired during passive listening to a rhythmic cue, without actually performing taps, and was established as a stable steady state in which RT preceded the cue by about 50 ms.

### Preparation for Movements in Random vs. Rhythmic Conditions

We found that negative asynchrony evolved rapidly during rhythmic tapping, but the exact number of taps required for synchronization was dependent on the underlying rhythm, consistent with previous reports (Peters, 1989). The impact of rhythmic cues on behavioral parameters (i.e., RT) was accompanied by changes in low-level motor parameters (i.e., acceleration and EMG). The pre-tap variability of the acceleration signal decreased before the tap in rhythmic tapping compared to tapping in response to randomly presented auditory cues. This difference in variability was restricted to the pre-tap period and was not found during or after the tap. In addition, the pre-tap activity of both flexor and extensor muscles was more variable in the random condition than in rhythmic tapping, but at a time window earlier than the time window found for the acceleration profile. Earlier studies reported that the onset of muscle activity and its standard deviation increased during self-paced vs. externally cued rhythmic movements but made no reference to random tapping (Safranek et al., 1982; Thaut et al., 1991, 1992). Moreover, the standard deviation can increase with an increased mean level of EMG without affecting the CV of the signal. Finally, the movements previously examined included gait and elbow movements, which may involve a very different control mechanism than the finger tapping used in this study.

These results show that while the actual taps are executed through a single mechanism regardless of the auditory context, the preparation for the tap is greatly affected by the auditory context. Note that between taps, subjects were required to keep their fingers extended to activate extensor but not flexor muscles. The increased variability in muscle activity for both muscles is thus not simply an outcome of the increased muscle activation; rather, it reflects the noisy input shared by the two muscle sets. The fact that the acceleration profile also exhibited increased variability suggests that the two muscle sets received a common source of noise that thus cannot be averaged out.

### Rhythmic Tapping in the Absence of Direct Auditory Cueing

We found that in rhythmic conditions, even when the auditory cue is absent, the pre-tap variability of the acceleration profile and muscle activity was significantly reduced. This was shown when the auditory cue was completely absent (self-paced tapping) or when it existed but not as a tap-triggering signal (during syncopation trials). The finding strongly indicates that the decrease motor variability was independent of any auditory processing.

Syncopation tapping provides an interesting case study. It was shown to require greater attention to the task (Carson et al., 1999), and its performance was therefore considered to require the recruitment of additional neuronal populations above and beyond those engaged in cued rhythmic synchronization (Jantzen et al., 2002; Mayville et al., 2002). Nevertheless, it appears here that once subjects became synchronized, syncopation taps were produced in a manner similar to rhythmic taps. This similarity was apparent in the pre-tap reduction of motor variability as well the occurrence of short ITI (350 ms instead of 500 ms) that indicate a negative asynchrony. We therefore suggest that the enhanced cognitive load in the syncopation task resides in the process of generating the precise time frame required for task performance. Once this time frame is generated, task performance is maintained in a manner similar to rhythmic tapping.

### The Entrainment Process

Entrainment is the phase in which subjects “learn” the rhythm and shift from reactive to predictive tapping. During this process the variability of inter-tap-intervals decreases and the RT gradually becomes negative. Another indication that subjects learned the rhythm was the appearance of an “extra tap” at the end of the auditory sequence. This tap reflects the prediction made by the subject and indicates the existence of a stable time frame.

Here we showed that the process of entrainment (i.e., the evolvement of negative asynchrony) was also reflected in a gradual decrease of variability of the acceleration profile. This suggests that feedback from the periphery (muscle, joint and skin receptors) is used for the entrainment process and that this process modifies the motor output in a tap-to-tap manner. Previous studies (Aschersleben et al., 2001) have attempted to evaluate the contribution of proprioceptive feedback by using local anesthesia of the tapping finger or using subjects lacking proprioceptive and tactile sensibility (Billon et al., 1996). They found an increase in the negative asynchrony, a result which corroborates the assumption that peripheral feedback affects the process of acquiring the appropriate time frame, though the authors did not report any differences in the entrainment process (e.g., number of taps required for negative asynchrony).

Nonetheless, when subjects passively listened to rhythmic cues and joined the rhythm only when instructed, they exhibited negative synchrony after a single tap. This implies that a stable time frame can be created by merely listening to rhythmic cues, in the absence of movement-related feedback during the entrainment process. This result reinforces the assumption that motor action is not a necessary element for creating a stable time frame and that proprioceptive feedback from the periphery is not a prerequisite for adjusting the temporal frame of the motor plan.

### Implications for the Mode of Operation of the Motor System During Different Auditory Contexts

This study shows that changing the tapping protocol from random to rhythmic affects motor output during preparation for movement but not during the actual movement. This means that timing of actions (which differs between random and rhythmic tapping) has low-level traces, such as reduced variability of muscle activity in the pre-tap period. This may further suggest that the timing of actions may not be independent of motor production and the processes through which actions are rhythmically timed have access to low-level motor parameters such as EMG and acceleration of movement.

**FIGURE 8:**
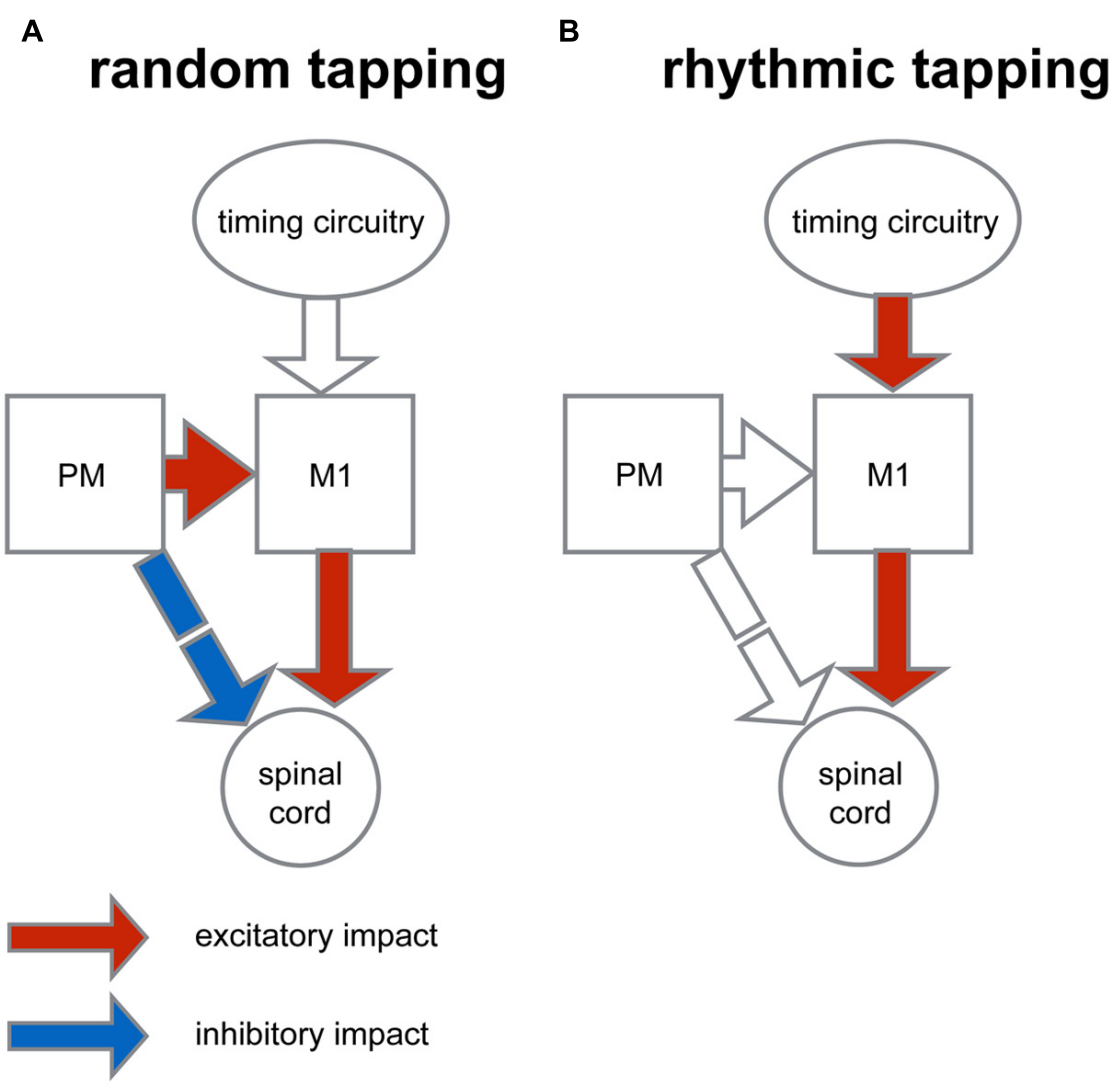
**Conceptual model for context-dependent motor preparation**. The figure illustrates a simplified organization scheme that describes the context-dependent differences in converging inputs onto spinal circuitry. **(A)** In preparation for random tapping premotor cortex (PM) exerts excitatory impact on the primary motor cortex (M1) and indirectly, an inhibitory impact on spinal circuitry (via the reticular formation). M1, in turn, exerts excitatory impact on spinal cord. This dual impact results in an excitation-inhibition balance during motor preparation at the spinal level which is translated into increased variability of motor actions. **(B)** During rhythmic tapping the movement timing is predicted and dictated by an alternative timing circuitry. This timing protocol obliterates motor preparation by PM activity, so that at the level of spinal circuitry activity balance is biased toward excitation and movement variability is reduced.

A conceptual model which could account for the results of this study is illustrated in **Figure 8**. In this model the increased motor variability measured in the random condition may be the outcome of a balanced inhibition-excitation state at the spinal level (Prut and Fetz, 1999; Cohen et al., 2010). According to this model, the unexpected nature of random tapping produces enhanced motor preparation. The preparatory activity which is dominant in the premotor cortex produces tonic excitation (Weinrich and Wise, 1982) which is fed to the spinal circuitry via two pathways (**Figure 8A**): a predominantly excitatory pathway relayed via the primary motor cortex through which spinal neurons receive specific movement-related commands, and a second inhibitory pathway, mediated by the reticular formation which provides global inhibition to prevent a premature release of the planned motor action (Moll and Kuypers, 1977). The balanced state produced by this “priming and breaking” mechanism during motor preparation is expected to yield increased variability (van Vreeswijk and Sompolinsky, 1996; Svirskis and Hounsgaard, 2003). In line with this reasoning, during rhythmic tapping, the preparatory period at the output level is no longer balanced (**Figure 8B**), leading to reduced variability which subsequently increases the precision of movement timing. One possible explanation for this change of state is that during rhythmic action the motor system operates in a mode where action onset is not explicitly triggered by the auditory cue (since taps occur slightly before the triggering signal); rather, an alternative circuitry could dictate the onset of actions onset. For example, it is possible that in rhythmic finger tapping, where the timing of action is presumably automatic the cerebellum provides the timing signal (Buhusi and Meck, 2005). Nonetheless, the timing of actions in response to rhythmically occurring cues could be dictated via alternative circuitry such as the cortico-thalamic-basal ganglia system (Merchant et al., 2013) or the prefrontal cortical areas (Laubach et al., 2015). This difference in timing protocol may obviate the need for cortical preparatory activity. Consequently, the underlying mechanism for producing motor action may be similar in all the auditory conditions of the tapping task, and the main difference between the forms of rhythmic actions would be the time needed for the internal timing circuitry to generate the appropriate time frame (i.e., the process of entrainment). This view contrasts with other models which have posited that different rhythmic tasks (e.g., syncopation) are mediated by different neural circuitries (Mayville et al., 2002).

## Author Contributions

MYF design the study, run the experiment, performed the data analysis, and wrote the manuscript. MA helped design the study and run part of the experiments. YP helped design the study, assisted with the data analysis, and participated in writing of the manuscript. All authors approved the final version of the manuscript.

## Conflict of Interest Statement

The authors declare that the research was conducted in the absence of any commercial or financial relationships that could be construed as a potential conflict of interest.
